# Constructing biomimetic liver models through biomaterials and vasculature engineering

**DOI:** 10.1093/rb/rbac079

**Published:** 2022-10-12

**Authors:** Weikang Lv, Hongzhao Zhou, Abdellah Aazmi, Mengfei Yu, Xiaobin Xu, Huayong Yang, Yan Yan Shery Huang, Liang Ma

**Affiliations:** State Key Laboratory of Fluid Power and Mechatronic Systems, Zhejiang University, Hangzhou 310058, China; School of Mechanical Engineering, Zhejiang University, Hangzhou 310058, China; State Key Laboratory of Fluid Power and Mechatronic Systems, Zhejiang University, Hangzhou 310058, China; School of Mechanical Engineering, Zhejiang University, Hangzhou 310058, China; State Key Laboratory of Fluid Power and Mechatronic Systems, Zhejiang University, Hangzhou 310058, China; School of Mechanical Engineering, Zhejiang University, Hangzhou 310058, China; The Affiliated Stomatologic Hospital, School of Medicine, Zhejiang University, Hangzhou 310003, China; School of Materials Science and Engineering, Tongji University, Shanghai 201804, China; State Key Laboratory of Fluid Power and Mechatronic Systems, Zhejiang University, Hangzhou 310058, China; School of Mechanical Engineering, Zhejiang University, Hangzhou 310058, China; Department of Engineering, University of Cambridge, Cambridge CB2 1PZ, UK; State Key Laboratory of Fluid Power and Mechatronic Systems, Zhejiang University, Hangzhou 310058, China; School of Mechanical Engineering, Zhejiang University, Hangzhou 310058, China

**Keywords:** biomaterials, extracellular matrix, liver, vasculature engineering

## Abstract

The occurrence of various liver diseases can lead to organ failure of the liver, which is one of the leading causes of mortality worldwide. Liver tissue engineering see the potential for replacing liver transplantation and drug toxicity studies facing donor shortages. The basic elements in liver tissue engineering are cells and biomaterials. Both mature hepatocytes and differentiated stem cells can be used as the main source of cells to construct spheroids and organoids, achieving improved cell function. To mimic the extracellular matrix (ECM) environment, biomaterials need to be biocompatible and bioactive, which also help support cell proliferation and differentiation and allow ECM deposition and vascularized structures formation. In addition, advanced manufacturing approaches are required to construct the extracellular microenvironment, and it has been proved that the structured three-dimensional culture system can help to improve the activity of hepatocytes and the characterization of specific proteins. In summary, we review biomaterials for liver tissue engineering, including natural hydrogels and synthetic polymers, and advanced processing techniques for building vascularized microenvironments, including bioassembly, bioprinting and microfluidic methods. We then summarize the application fields including transplant and regeneration, disease models and drug cytotoxicity analysis. In the end, we put the challenges and prospects of vascularized liver tissue engineering.

## Graphical Abstract

**Figure:**
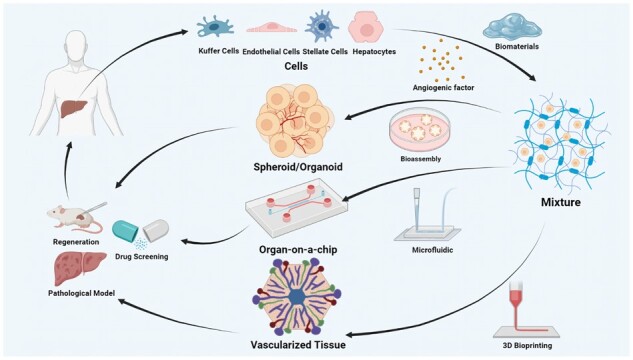

## Introduction

The liver is the largest gland in the human body and is responsible for a variety of critical biological functions in the body, including the metabolism of carbohydrates, proteins and lipids, bile production and detoxification [1]. It is well established that the liver has a strong regenerative capacity. However, liver fibrosis, viral infection and drug damage will reduce this regeneration ability and cause irreversible damage [2]. Hence, the manufacture of complex liver tissues with adequate functionality has become particularly important due to the high demand for organ transplantation and the inability to replace *in vitro* models required for new drug development and organ pathology research [3, 4]. Liver tissue engineering is considered the most promising alternative to mimic microstructure and maintain major function for liver implantation and drug screening [5–8]. Three-dimensional (3D) hepatic cell culture can generate complex cell structures, shapes and arrangements, which significantly increases the accuracy and reliability of experiments. It has significant advantages in constructing the heterogeneity and complexity of liver tissues. In order to culture hepatocytes *in vitro* for a long time and fully reproduce the liver functionalities, it is of great importance to reconstruct the unique liver vasculature system [9, 10].

Under physiological conditions, the human vasculature has essential biological functions, such as nutrient and gas exchange, metabolic waste removal and homeostasis maintenance. The diffusion limit of oxygen and nutrients is about 200 μm, which means that cells farther from the capillaries experience hypoxia and apoptosis [11–13]. The liver is one of the most vascularized organs in the body. Therefore, the constructed vascularized liver tissue will more favorably mimic the physiologically heterogeneous structure and cellular microenvironment, which can make it more convenient for biomedical applications in regenerative medicine and drug development [14].

Hepatic extracellular matrix (ECM) is a complex macromolecular network that not only provides cells with a natural microenvironment but also participates in the regulation of cellular functions [15, 16]. For example, the regulation of cell motility and differentiation is controlled by the surrounding physical environment and biochemical signals [17]. Biomaterials should then be able to recreate the key features of the extracellular microenvironment, including microarchitecture, mechanical strength, tissue-specific protein composition and pro-angiogenic properties to maintain cell morphology and growth [17–20]. Different types of biomaterials, which can be broadly classified into natural hydrogels and synthetic polymers, have been used in liver tissue engineering to reconstruct the liver ECM [21, 22].

Another important factor in liver tissue engineering is the cell sources. According to the differentiation degrees of cells, the fabrication methods for adapted liver tissues are also different. Undifferentiated cells have good biological activity and differentiation potential, and the co-culture of cells allows communication through paracrine factors and contributes to the formation of complex cellular structures with more functionalities [23–26]. By combining with highly biocompatible biomaterials, the microenvironment is used to control cells for the differentiation and regeneration of blood vessels. These printed liver tissues can deposit 3D microstructures more accurately by using extrusion or photocuring to build complex vascular networks with fixed patterns [27–29]. Combining biomaterials with stronger mechanical properties can ensure the stability of the spatial structure and increase the tightness of the connection between cells. In addition, the microfluidic-based organ-on-a-chip technology has the characteristics of dynamic perfusion culture [30, 31]. Nutrients and metabolites flow through the internal microchannels and diffuse between endothelial cells and hepatocyte layers, simulating the physiological microcirculation.

**Figure 1.**
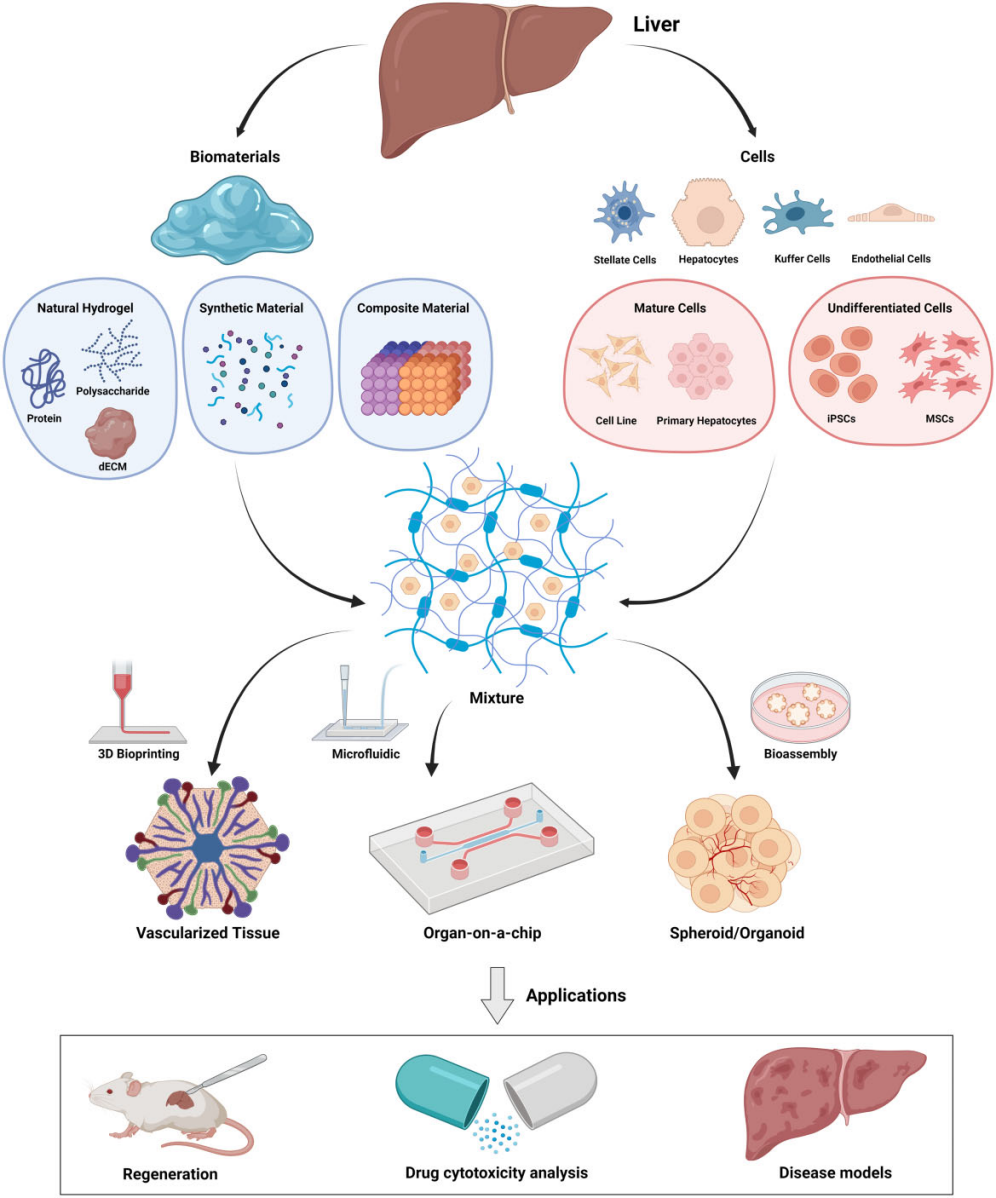
Schematic of vascularized liver tissue engineering (created with BioRender.com).

In this review, we discuss recent advances in tissue engineering of vascularized liver tissues, with a special focus on the biomaterials and cells matched to different liver extracellular matrices. In summary, we first describe the structure and function of the hepatic vasculature and then classify applicable biomaterials. Based on the degree of cell differentiation, it is roughly divided into differentiated cells and mature cells, thereby developing different strategies for constructing specific vascular systems *in vitro*. Then, the applications of the prepared vascularized liver tissues are discussed, such as drug discovery, disease model and liver regeneration. Finally, the current challenges and future perspectives of vascularized liver tissue engineering will be provided. The schematic of vascularized liver tissue engineering is summarized in Fig. 1.

## Structure of the liver

The liver is the largest gland in the human body with complex and diverse biochemical functions [32]. At the same time, the liver is rich in blood supply, the liver vascular system includes the inflow and outflow blood vessels, the former refers to the proper hepatic artery and the portal vein, and the latter refers to the hepatic vein [32]. After entering the liver, both the hepatic artery and portal vein branch repeatedly and become thinner and thinner, finally form interlobular arteries and interlobular veins, which pass through the hepatic plate and connect with the hepatic sinusoids to form a rich vascular network. Subsequently, the central vein of the hepatic lobule merges into the inferior lobular vein, which finally merges into the hepatic vein [33].

The unique feature of the liver vasculature is the dual blood supply. Its blood supply includes the portal vein and the hepatic artery. The portal vein mainly collects the blood flow of the gastrointestinal tract and the splenic vein, and transports nutrients and some toxic substances to the liver for metabolism, and the hepatic artery mainly provides oxygen [34].

**Figure 2.**
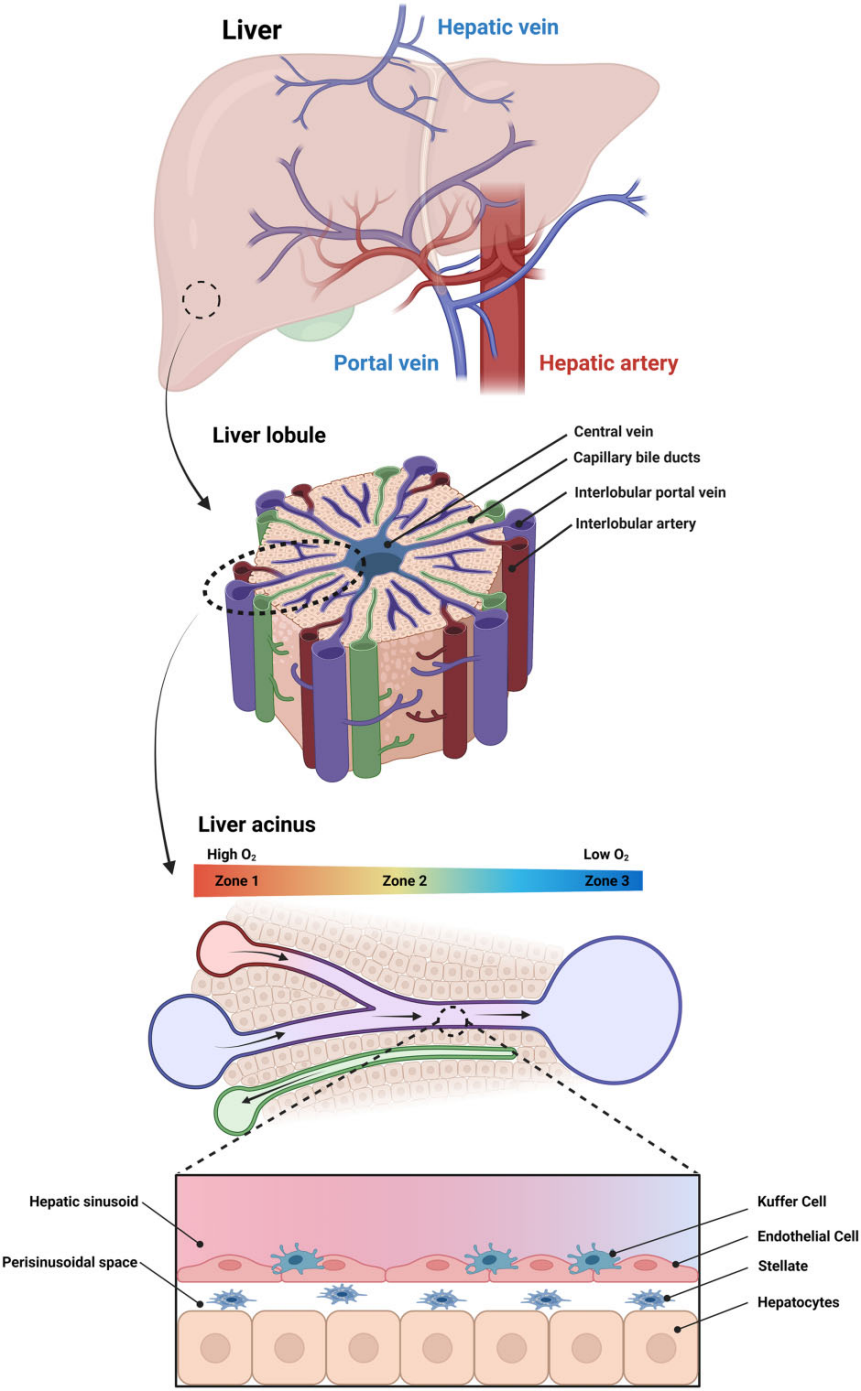
Structure and elements of the liver (created with BioRender.com).

Although the normal liver is mainly composed of parenchymal cells, the surrounding fibrous tissue is very limited in number, the extracellular microenvironment has significant effects on the physiological organization and organ function [17, 35]. Therefore, the ECM plays a crucial role in liver physiology and pathology, and any modification of the ECM will directly impact liver function [36]. The structure and elements of the liver can be seen in Fig. 2.

### Basic units of the liver

Due to the different perspectives of studying the relationship between the structure and function of the liver, the essential components can be divided into two types: hepatic lobule and hepatic acinus [37].

#### Hepatic lobule

The hepatic lobules are polygonal. It has a central vein that runs along its long axis and is surrounded by radially arranged hepatic cords and sinusoids [38, 39]. Hepatocyte monolayers are arranged in an uneven plate-like structure called the liver plate. Adjacent liver plates are anastomosed and connected to form the hepatic cords [39]. The hepatic sinusoids are located between the hepatic plates and communicate with each other through pores in the hepatic plates, forming the smallest capillaries of the liver [40]. The plasma membrane on the adjacent side of the hepatocytes is partially recessed, forming tiny bile ducts. In this way, the hepatic plate, hepatic sinusoids and bile ducts form an independent and closely related complex network within the hepatic lobules [33]. The area between adjacent hepatic lobules is called the portal area. There are three to six portal areas around each hepatic lobule, in which three accompanying ducts can be seen, namely interlobular veins, interlobular arteries and interlobular bile ducts [37].

##### Hepatic sinusoids

The hepatic sinusoids are located between the hepatic plates, the cavity is large and irregular, and the sinus wall is surrounded by endothelial cells [41]. The blood from the interlobular arteries and the interlobular veins flows into the hepatic sinusoids [42, 43]. Due to the slow blood flow in the sinusoids, the plasma can have sufficient material exchange with the liver cells and then flow into the central vein [44, 45].

##### Perisinusoidal space

The perisinusoidal space is the narrow space between the hepatic sinusoidal endothelium and the hepatic plate, also known as the Disse space, where substances exchange between liver cells and blood [44]. Due to the high permeability of the hepatic sinusoid endothelium, the perisinusoidal space is filled with plasma, and a large number of microvilli on the blood perisinusoidal surface of the liver cells are soaked in the plasma, which can conduct sufficient and efficient material exchange [46].

#### Hepatic acinus

The hepatic acinus is smaller in size and resembles the shape of an olive [47]. It is a substantial block with the terminal branch of the portal canal as the central axis and the central vein as the boundary at both ends [48]. The blood flows from the portal area of the central axis into the interlobular vessels and passes through the hepatic plate to connect with the hepatic sinusoids. After sufficient material exchange, blood flows radially to the central veins at both ends, forming a complex anastomotic vascular network. Blood flow along the radial axis of the leaflet creates a gradient of oxygen, nutrients and hormones, creating a highly variable microenvironment [48–50]. According to the direction of blood flow and the order of nutrient acquisition, the hepatic acinus can be divided into three regions [51–54]. The periportal hepatocytes around the afferent vessels and the hepatocytes around the central vein around the efferent vessels show different metabolic capacities and subcellular structures, resulting in the metabolic division of hepatic acinus [52]. Hepatic microcirculation is closely related to the pathogenesis of pathology, and the metabolic zoning of hepatic acini can reasonably explain the pathogenesis of some liver lesions [6, 51, 55, 56].

### Extracellular matrix of the liver

The ECM constitutes a complex protein network that enables tissue to form an integral structure and plays an essential role in regulating tissue homeostasis, remodeling and regeneration [21, 57, 58]. Its mechanical elastic properties can support cells to maintain their shape and structure, and the excellent biological properties provide a microenvironment that promotes cell survival, proliferation, differentiation and migration [16, 58]. In normal liver, the ECM occupies ∼3% of the relative area and 0.5% of the weight. Collagen types I, III, IV and V are the most abundant proteins in the intrahepatic matrix [59, 60]. Other components of the hepatic ECM are present in small amounts, mainly including glycoproteins such as laminin, fibronectin, tenascin, nestin and SPARC [59]. Proteoglycans include heparan, dermatan, chondroitin sulfate, perlecan, hyaluronic acid (HA), biglycan and decorin [60].

#### ECM of hepatocytes

Type I collagen and fibronectin are mainly found around hepatocytes. A small amount of ECM is better for cell adhesion and improves the mechanical coherence and resistance of the liver [60]. Meanwhile, the liver ECM also plays a role in several major biological functions such as cell proliferation, migration, differentiation and gene expression [36].

#### ECM of the vasculature

For the ECM of the vasculature in the hepatic lobules, laminin and collagen IV surround the bile duct, and collagen types I, III and V are mainly confined to the walls of the portal and central veins [15, 46]. Collagen type IV binds to laminin and entactin/nidogen to form a basement membrane-like substance along the sinus walls [36]. The low density of the ECM, accompanied by the abundant perisinusoidal endothelial cell windows, is ideal for facilitating the rapid bidirectional exchange of macromolecules that occurs between blood and hepatocytes, which is also critical for maintaining the differentiation of adjacent hepatocytes [17].

#### ECM in lesions

ECM also plays a significant role in pathological liver models, such as liver fibrosis. The composition of ECM is similar to that in normal liver, but the relative amount of the components is redistributed, and the amount increases to three to five times [35]. The most obvious change is the dilation of the ECM from the portal or central veins [61]. Specifically, type I collagen can better reflect the degree of liver fibrosis, and type III collagen is positively correlated with the degree of liver cirrhosis and is more closely related to liver inflammation. Type IV collagen is an essential component of the basement membrane. In the early stage of liver fibrosis, type IV collagen hyperplasia can form a basement membrane in the Disse space [21, 62, 63]. Although fibrosis is a major biological event, it is inextricably linked to other important mechanisms in the liver, such as hepatocyte regeneration and vascular redistribution.

## Cells and biomaterials for vascularized liver tissue

Mixtures of cells and biomaterials are mainly used to construct liver tissue *in vitro*. Among them, cells mainly play the role of biological functions, while biomaterials can promote cell growth and maintain the shape of the overall structure.

### Cell types and sources

The liver is mainly composed of hepatic parenchymal cells and hepatic non-parenchymal cells. Liver parenchymal cells are mainly hepatocytes, which perform the major metabolic and protein secretion functions of the liver. Non-parenchymal cells include Kupffer cells, endothelial cells and hepatic stellate cells (HSCs), which are mainly used to support hepatocytes and improve the functional structure of the liver [64]. In this section, we will introduce cells for 3D bioprinting according to these categories. Table 1 summarizes the main cell types and sources used in 3D bioprinting.

**Table 1.** Cell type and source

Cell type	Cell source	Functionality	References
Hepatocytes	Primary hepatocytes, from human	Metabolic and secretory functions of the liver	[65, 66]
	HepG2 (hepatocellular carcinoma, cell line, from human)	Metabolic and secretory functions of the liver	[67–70]
	Huh7 (hepatocellular carcinoma, cell line, from human)	Metabolic and secretory functions of the liver	[71, 72]
	HepaRG (hepatic progenitor cells, from human)	Can differentiate into hepatocytes and bile duct cells to achieve liver metabolism and secretion	[73, 74]
	MSCs (mesenchymal stem cells, from human)	Has the potential to differentiate into a variety of cells, it can differentiate into hepatocytes for metabolism and secretion	[75–77]
	hiPSCs (human-induced pluripotent stem cells, from human)	Has the potential to differentiate into a variety of cells, it can differentiate into hepatocytes for metabolism and secretion	[25, 26, 78–80]
Kupffer cells	Primary Kuffer cells, from human	Remove foreign bodies, monitor tumors	[66]
	Kup5, (c-myc-immortalized Kupffer cells, cell line, from mouse)	Remove foreign bodies, monitor tumors	[81]
Stellate cells	LX-2 (hepatic stellate cells, cell line, from human)	Involved in vitamin A metabolism and fat storage, producing extracellular matrix	[82]
Liver perisinusoidal endothelial cell	HUVEC (human umbilical vein endodermal cell, cell line, from human)	Reduce blood flow rate, promote substance interaction	[14, 81, 83]
Cholangiocytes	SV40SM (cholangiocytes, cell line, from mouse)	Formation of bile ducts	[84]
	Cholangiocarcinoma cells, from human	Induced hepatocarcinogenesis	[85]

#### Hepatocytes

Liver parenchyma cells are the most numerous and densest cell population in the liver, accounting for 80% of the total number of total liver cells [2, 86]. There are well-developed villi on the surface of the blood sinus and bile canaliculi of hepatocytes, which increase the surface area of the cells and promote the exchange of substances [87].

Such cells perform major hepatic metabolic and secretory functions. Primary cells have high metabolic activity and are the ideal cell source [86, 88, 89]. However, due to the lack of human primary hepatocytes, these cells can easily lose their phenotype, so the *in vitro* tissue construction based on primary hepatocytes is still difficult. To meet the research needs, researchers have developed a variety of cell lines with good proliferation and characterization. It is worth mentioning that although HepG2 are tumor cells, their expression functions are roughly consistent with those of normal liver cells [29]. At the same time, it has the ability of hypoxic respiration and high proliferation that normal liver cells do not have. Therefore, in previous research, these cells have been widely used in 3D bioprinting to construct disease models and test drug cytotoxicity [14, 28, 67, 68]. However, researchers are still working to improve liver tissue function *in vitro*. Since some progenitor cells or pluripotent stem cells can differentiate into fully functional hepatocytes *in vitro*, they have unparalleled advantages in constructing perfect structure and protein expression [90]. For example, HepaRG cells, which retain bipotent hepatic progenitor-like characteristics, are the most promising type for bioprinting to construct *in vitro* liver tissue [73, 74, 82]. Therefore, cells of this type are increasingly used in the latest research.

#### Endothelial cells

Endothelial cells have numerous fenestrations of varying sizes [91, 92]. Endothelial cells are loosely connected with wide intercellular spaces [93, 94]. There is no basement membrane outside the endothelium, and only a small amount of reticular fibrin is attached [91, 95]. Therefore, the hepatic sinusoidal endothelium has a high permeability, and various plasma components can enter the perisinusoidal space [93, 96–99]. In most research, hepatic sinusoidal endothelial cells can be replaced with human umbilical vein endothelial cells (HUVECs) [100–102]. This type of cell grows faster, can spread efficiently, and spontaneously induce directional induction based on endothelial growth factors to form pathways [103, 104]. Therefore, it is very suitable for building vascularized structures.

#### Kupffer cells

Liver sinusoids contain resident macrophages that attach to endothelial cells and can penetrate endothelial fenestrations and intercellular spaces deep into the peri-sinusoidal space [92, 105]. Due to the active phagocytic ability of cells, they play an important role in clearing the liver of antigenic foreign bodies, senescent blood cells, and monitoring tumors [97, 106, 107]. In the uninjured liver, Kupffer cells constitute the major population of inflammatory cells and are important for many homeostatic functions [108, 109].

#### Hepatic stellate cells

There are irregular fat-storing cells in the perisinusoidal space, also known as HSCs, which are mainly involved in the metabolism of vitamin A and the storage of fat in the liver [110, 111]. In addition, another function of HSCs is to produce ECM, from which the reticular fibers in the perisinusoidal space are produced [112–114]. Under pathological conditions, adipocytes are activated and abnormally proliferate, producing ECM and increasing intrahepatic fibrosis, which can finally lead to cirrhosis [110, 115, 116].

### Interaction between hepatocytes and endothelial cells

The reciprocal relationship between the development of the liver and the vasculature has long been investigated and elucidated [117]. Early research studies questioned the fast regeneration of the liver, which pushed them to use partial hepatectomy models removed generally from rats [117]. These hepatectomy models allowed the discovery of reciprocal communication between liver sinusoidal endothelial cells (LSECs) and hepatocytes using vascular endothelial growth factor (VEGF) and hepatocyte growth factor (HGF) factors [118].

HGF was later found as the most potent simulator of hepatocytes which is produced by LSECs and HSCs. The binding of HGF and the tyrosine kinase receptor c-Met consequently activates several signaling pathways, including JAK/STAT3, phosphoinositide 3-kinase (PI3K)/Akt/NF-κB, Ras/Raf and mitogen-activated protein kinase (MAPK) cascades [119]. Each of these pathways has a specific role in liver regeneration, such as regulating cell growth, migration, differentiation and apoptosis [119]. The HGF/c-Met pathway has a vital role in maintaining a normal liver indeed. However, slight dysfunction of this pathway may cause diverse pathologies such as tumors [120, 121]. On the other hand, VEGF is the main glycoprotein responsible for vascular growth, including vasculogenesis and angiogenesis [98]. In the liver, the VEGF is produced by hepatocytes and HSCs [98].

Hepatectomy models have served as great proof of the crucial role that plays the endothelial cells in the liver. Therefore, the co-culture of endothelial cells and hepatocytes should offer a more accurate *in vitro* liver model, which has indeed been approved consequently. In an interesting, Wang *et al*. [122] used a co-culture system containing hepatocytes and ECs with different ratios. As a result, this co-culture system showed to improve the secretion of albumin and urea and the expressions of albumin, BYP3A4 and HNF4α. These findings have also been enhanced by Lee and Cho [123] using a more sophisticated dynamic liver model. The research team have used a one-step microextrusion-based bioprinting method to create a PCL-based liver-on-a-chip model. A dynamic co-culture model of hepatocytes and endothelial cells was shown to provide greater albumin secretion and urea synthesis with higher cell viability.

### Biomaterials

**Figure 3.**
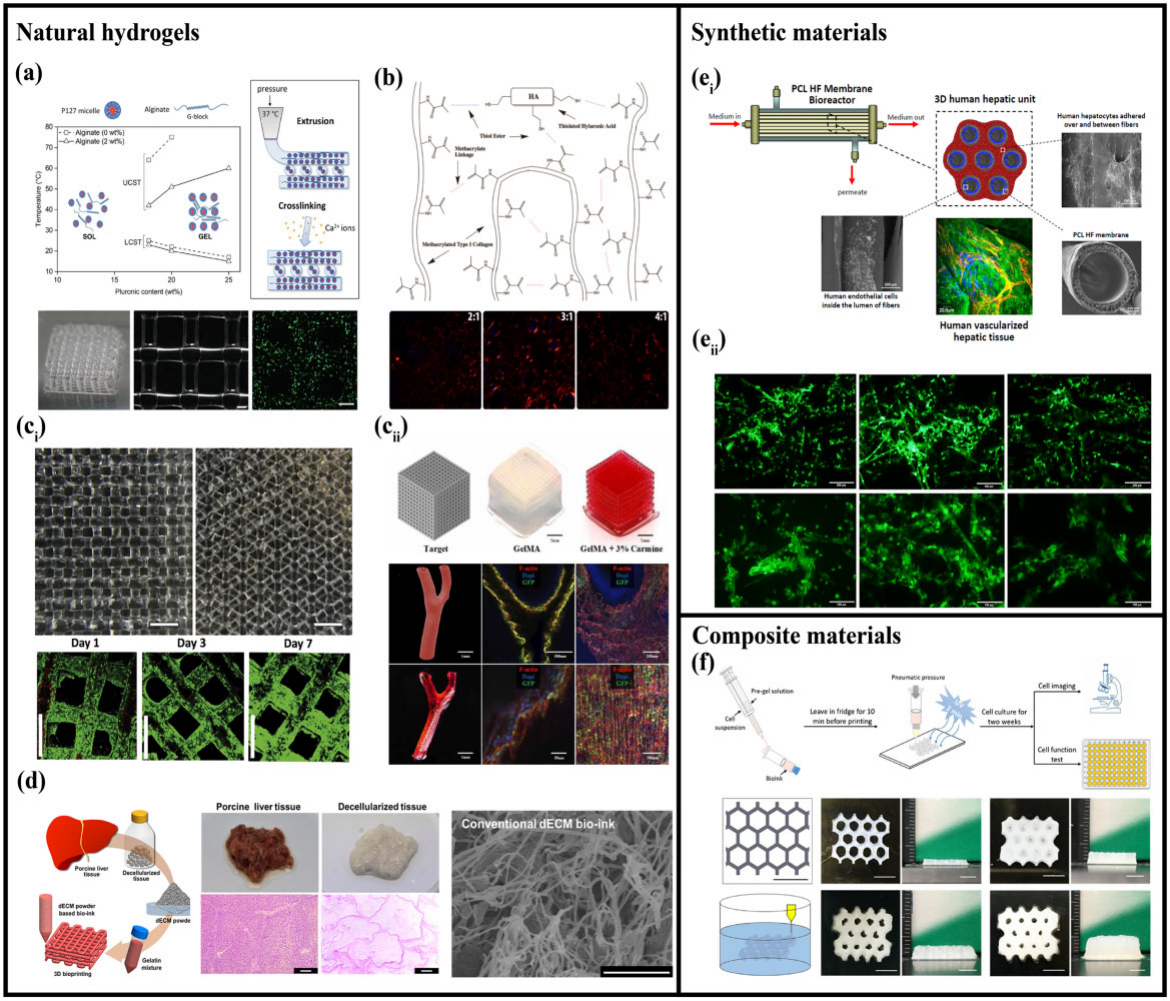
Biomaterials used in the vascularized liver tissue engineering. (**a**) Pluronic and alginate with high applicability. Adapted with permission from Ref. [140]. (**b**) Collagen type I with hyaluronic acid. Adapted with permission from Ref. [149]. (**c**) Gelatin. (**c_i_**) Gelatin scaffolds. Adapted with permission from Ref. [71]. (**c_ii_**) Photocrosslinking of GelMA, producing vascularized tissues with complex shapes. Adapted with permission from Ref. [147]. (**d**) dECM bioink with enhanced printability and mechanical properties. Adapted with permission from Ref. [151]. (**e**) Synthetic polymers. (**e_i_**) PCL mixed with biodegradable hollow fibers (HF). Adapted with permission from Ref. [156]. (**e_ii_**) PLGA scaffolds. Adapted with permission from Ref. [157]. (**f**) Cellulose nanocrystals (CNC). Adapted with permission from Ref. [164].

Given that hepatocytes are anchorage-dependent cells and the ECM is required for their survival and functionality realization [124]. Therefore, in past studies, different kinds of biomaterials have been investigated for their successful cell cultures. By examining the cellular morphology and characterization of hepatocytes used in 3D bioprinted *in vitro* models, different biomaterials can be developed to mimic as much as possible the *in vivo* microenvironment as well as the physiological changes of cells *in vitro*, thus closer to the actual situation. The commonly used biomaterials for the construction of vascularized liver tissue are summarized in Fig. 3.

#### Natural hydrogels

Hydrogels are water-swellable, highly cross-linked polymer networks that are soft, quasi-solid, and can support and protect cells. Since protein-based and polysaccharide-based ECM occupies most of the liver, this type of hydrogel has a significant promoting effect on the cell growth of liver tissue *in vitro* and is more conducive to restoring the cellular microenvironment *in vivo* and improving the metabolism effect of the liver cells, is commonly used to prepare scaffolds for liver regeneration. Table 2 summarizes some of the commonly used natural hydrogels.

**Table 2.** Normal natural hydrogels for liver tissue engineering

Type	Origin	Constituents	Crosslinking	Fabrication	Features	References
Alginate	From cell walls of algae/seaweed	Guluronic acid and mannuronic acid	Ionic (Ca^2+^, Ba^2+^, Mg^2+^)	Extrusion-based bioprinting; Inkjet-based bioprinting	With shear thinning properties, abundant sources, easy to crosslink	[125–127]
Agarose	Marine seaweed	3,6-anhydro-L-galactopyranose and D-galactose	Temperature	Extrusion-based bioprinting	High propensity to gelation	[128]
Chitosan	Insects and crustacean exoskeleton	N-deacetylation of chitin and glycosaminoglycans	Ionic and covalent cross-link	Extrusion-based bioprinting	High viscosity fluids, resistant to bacteria and fungi and analgesia	[129, 130]
Hyaluronic Acid (HA)	ECM, all over the body	β1, 4D glucosamine and N-acetyl-D-glucosamine	Physical, light-controlled chem	Extrusion-based bioprinting	Hydrophilicity and biocompatibility, faster healing	[131]
Gelatin	Cow skin, pig skin, fish skin	Glycosaminoglycans	Covalent, enzymatic, temperature, physical	Extrusion-based bioprinting	It is sensitive to thermal environment, effective cell bonding	[132]
Gelatin MA	Semi-natural polymer	Gelatin and acrylates	Light (UV)	Digital light processing (DLP)-based 3D bioprinting; Extrusion-based bioprinting; Inkjet-based bioprinting	Facilitate attachment of cells with scaffold promoting adhesion and cell activity, have great mechanical properties	[133, 134]
Collagen	Nature-derived ECM, the tendon of rat tail, fish skin	Chains of polypeptide	pH, ionic cross-link, temperature, protein riboflavin	Extrusion-based bioprinting; Inkjet-based bioprinting	Enzyme catalyzed degradation, poor printability	[135, 136]
Fibrin	Distributed in plasma	Fibrous and non-globular glycoproteins	Enzymatic Crosslinking (Factors IIa, XIIIa and IV)	Extrusion-based bioprinting	Biodegradable with cell adhesion sites	[137, 138]
Decellularized extracellular matrix (dECM)	Extracellular matrix	Collagen, fibrin, gelatin	Light (UV), enzymatic	Digital light processing (DLP)-based 3D bioprinting; Extrusion-based bioprinting;	With complex natural ingredients and biomimetic structure	[139]

##### Alginate

Alginate has been widely used in liver tissue engineering due to its good biocompatibility, abundant sources, easiness to cross-link and low price [126, 127]. Gori *et al*. [140] developed an *in vitro* 3D liver model using a composite hydrogel of the biologically inert materials Pluronic and alginate with high applicability in supporting the viability and metabolic activity of the HepG2 cell line (Fig. 3a). Taymour applied alginate and methylcellulose (algMC)-based bioinks for core-shell bioprinting and established a functional co-culture model with independently tunable compartments for different cell types which provides a liver-like microenvironment for more complex *in vitro* models [141]. However, pure alginate does not provide a cell adhesion site for the encapsulated cells. Researchers usually modified the alginate hydrogel with some cellular recognition motifs such as Arginylglycylaspartic (RGD), which is widely used in the engineered hydrogels to bind the cell adhesion protein integrins, hence significantly enhancing the cell adhesion and proliferation [142].

##### Hyaluronic acid

HA is the main component of the peri-sinusoidal space and has excellent biocompatibility and biodegradability, which plays an important role in cell proliferation and angiogenesis [131]. As in the process of liver fibrosis, HSCs are activated, resulting in the massive production of a hard matrix. In order to study the cellular mechanotransduction of HSCs in related diseases, Caliari *et al*. [143] used HA to prepare a hardened hydrogel model to simulate liver cirrhosis and proved the stiffening HA hydrogels could be a more faithful model for studying myofibroblast activation than traditional static substrates.

##### Gelatin

Gelatin and its derivatives are another widely used hydrogels for the construction of livers *in vitro* [132]. The triple helical structure of collagen can be lost by hydrolysis, so gelatin is more soluble in a hydrophilic medium and more convenient to prepare [144]. Lewis *et al*. [71] demonstrated the ability to precisely control the pore geometry of 3D printed gelatin scaffolds (Fig. 3ci). They showed high viability and proliferative capacity when seeded on 3D printed scaffolds of different geometries with an undifferentiated hepatocyte cell line (HUH7). However, due to the long cross-linking time and poor printability of gelatin, researchers gradually eliminated it in the fabrication of precise microstructures.

Gelatin Methacrylamide (GelMA) is a modified gelatin that crosslinks in seconds under UV light [133, 145]. Compared with other photocurable hydrogels, GelMA is widely favored by researchers due to its excellent biocompatibility and strong mechanical properties at the same time [146]. Sun *et al*. [147] precisely controlled the degree of photocrosslinking of GelMA, producing vascularized tissues with complex shapes, high precision, and controllable mechanical properties (Fig. 3cii). Roopesh *et al*. [148] made sandwiched liver parenchyma microtissues with GelMA. Monitoring of liver-specific function revealed that the 3D structure of liver tissue in the hydrogel sandwich was maintained while compared with it in suspension, albumin secretion, urea synthesis and CYP450 activity were enhanced. These researches all demonstrate the potential of GelMA for *in vitro* tissue construction.

##### Collagen

Collagen is the most abundant component in the liver ECM, especially type I collagen, but due to its low viscosity, collagen is less suitable for bioprinting [135]. Therefore, various methods are currently developed to improve the mechanical properties and printability of collagen. Mazzocchi *et al*. [149] printed 3D liver tissue structures containing primary human hepatocytes (PHHs) and HSCs using a mixture of collagen type I and HA and found that hepatocytes were able to express albumin and survive for more than 2 weeks while responding appropriately to acetaminophen, a common liver poison (Fig. 3b). Deng *et al*. [74] seeded cells in microporous platforms to form spheroids, which were then directly encapsulated in mixed hydrogels containing various collagen and protein, including collagen type I (COL1), collagen IV (COL4), fibronectin protein (FN) and laminin (LM). The results showed that different ECM components promoted the expression and secretion of hepatic markers of cell spheroids [74].

##### Decellularized extracellular matrix

The natural ECM has better biological properties and degradability, which can provide a scaffold for a 3D liver microenvironment with complex natural components and biomimetic structures, which is necessary for the development of better tissue models [124, 150]. However, its poor printability and weak mechanical properties remain a challenge. Kim *et al*. [151], developed a new gelatin-mixed decellularized ECM (dECM) bioink with enhanced printability and mechanical properties (Fig. 3d). In order to further solidify the acellular ECM to strengthen its mechanical properties, Mao *et al*. [152] developed a liver-specific bioink and encapsulated human-induced hepatocytes (hiHep cells) to form cell-laden bioinks and found that hepatocytes spread farther in this microtissue and had better hepatocyte-specific functions. In the research of biocompatibility, Sharma *et al*. [72] developed a hybrid liver-specific three-dimensional scaffold using gelatin with native decellularized liver matrix (DCL) and silk fibroin, providing a favorable microenvironment for enhanced hepatocyte differentiation and function. Minami *et al*. [26] successfully developed a novel artificial liver model using human-induced pluripotent stem cells and rat decellularized liver scaffolds and demonstrated its potential to promote functions characteristic of human livers. It can be seen that the high biocompatibility of acellular ECM has great advantages in inducing undifferentiated cells to maintain cell-specific functions and form *in vitro* tissues.

#### Synthetic materials

Although natural biomaterials have absolute advantages in biocompatibility, some problems, such as low viscosity, poor mechanical properties and insufficient sources, hamper their biomedical applications. Synthetic polymeric with better mechanical properties have become increasingly used in liver tissue engineering, especially for vessel structures requiring precise and better shape retention. It has high mechanical strength, which can be applied to certain research purposes. Meanwhile, to overcome the low binding affinity for cells, these synthetic polymers are often modified with biomolecules (e.g. proteins, polysaccharides, polypeptides) to improve their biocompatibility by adding cellular recognition motifs. Among the many 3D printable synthetic polymers, polyethylene glycol (PEG), poly(ε-caprolactone) (PCL) and poly(lactic-co-glycolic acid) (PLGA) are primarily used for bioprinting of liver structures [153, 154]. They have tunable mechanical properties that can provide a microenvironment to guide liver regeneration and remodeling. PCL has good biocompatibility and processability, softness and flexibility at body temperature [144]. Lee *et al*. [155] injected a bioink containing primary hepatocytes, HUVECs, and human lung fibroblasts into PCL scaffolds to induce capillary-like network formation and hepatocyte growth. A co-culture 3D microenvironment of these three types of cells was successfully established and maintained [155]. Salerno *et al*. [156] reported a bioink based on PCL mixed with biodegradable hollow fibers. After printing the liver tissue model, it was found that endothelial cells were massively integrated with the inner surface of individual PCL fibers to form a blood vessel-like structure, and hepatocytes completely covered the outer surface and the space between the fibers (Fig. 3ei) [156]. Furthermore, the tunable degradability and support properties of PLGA can be adapted to the regeneration process. Liu *et al*. [157] co-cultured mesenchymal stem cells (MSCs) and hepatocytes and demonstrated the stable differentiation ability of MSCs upon hepatocytes in PLGA scaffolds (Fig. 3eii).

#### Composite/multicomponent/hybrid materials

Although a single hydrogel is easy to prepare, it still has limitations in its multifunctional biocompatibility with multiple cell types. Therefore, in order to further reduce the components of the ECM, composite/multicomponent/hybrid materials are gradually used for liver construction.

##### Hybrid

Hybrid materials are generally a mixture of multiple collagens or extracellular matrices. The diversity of this material composition has a positive effect on the growth of hepatocytes. Clark *et al*. [158] described a novel bioink composed entirely of materials in the ECM of human tissue. This was achieved by incorporating gelatin nanoparticles into a base bioink made of collagen methacrylated and HA, with excellent mechanical properties and printability. Matrigel is a biomaterial that regulates the cellular microenvironment and contains various components such as cohesin, collagen IV, fibronectin and heparan sulfate [159, 160]. Tao *et al*. [161] supplemented macromolecules including Matrigel and polysaccharides at different concentrations into HepG2 spheroids to modulate the cellular microenvironment and observed the effect on cell viability.

##### Nanocelluloses

In the context of biomedical hydrogels for tissue engineering, one particular kind of synthetic nanomaterials called nanocelluloses share structural similarities to the ECM due to their porosity and interconnected framework within the structural hydrogel [162]. Meanwhile, the fiber morphology of cellulose fibrils is somewhat similar to that of collagen and fibronectin, which has attracted great attention. This provides better shape fidelity and print resolution for the stent. Wu *et al*. [163] mixed alginate and cellulose nanocrystals to prepare a hybrid bioink with shear-thinning rheological properties. Fibroblasts and hepatoma cells were then cultured together on the printed scaffolds and found that the viability of the cells was not affected. Subsequently, they designed a new bioink using alginate, cellulose nanocrystals and GelMA, which can directly print cell-laden structures by micro-extrusion. The ink exhibits excellent shear thinning behavior and solid-like properties, enabling high printability without obvious cell damage (Fig. 3f) [164]. The composite bioink of cellulose nanofiber hydrogels combined with alginate is an effective method for achieving cross-linking of printed scaffolds in the presence of Ca^2+^. The porous structures formed can help cells adhesion on the surface and inside of the scaffolds to improve cell viability. Zhang *et al*. [165] prepared a CNF-alginate-CLP (cellulose nanofiber hydrogel-alginate-spherical colloidal lignin nanoparticle) nanocomposite scaffold to which CLPs brought antioxidant properties and increased the viscosity of the hydrogel at low shear rates, HepG2 cells encapsulated remain high cell viability proved that this kind of nanocomposite is suitable for liver tissue engineering.

## Biomanufacturing approaches for vascularized liver tissues

Various new strategies for vascularized liver tissue creation are being proposed as biomanufacturing technology advances. From the level of cell origin, it can be simply divided into two categories. One is progenitor cells or stem cells with differentiation potential. The majority of methods employing such cells for liver tissue development use scaffold-free approaches to generate spheroids or organoids without fixed structures and rely on cells’ differentiation ability to stimulate the creation of blood vessels. The other is mature cells, which do not have the ability to differentiate. Most of them use 3D bioprinting methods to construct hepatic lobular tissue with vascular structure or microfluidic-based methods to prepare liver-on-chips [166]. In the following paragraphs, we will briefly discuss different manufacturing strategies for using these two types of cells to construct vascularized liver tissue *in vitro*.

### Bioassembled vascularized liver models

Organoids constructed from undifferentiated cells have the ability to self-renew, are closer to real tissues, and have highly similar histological functions. In general, strategies for vascularizing spheroids and organoids are carried out in two steps: First, organoids are constructed *in vitro*, and cells are induced to differentiate to form primary blood vessels. Then, transplantation into highly vascularized regions of the liver *in vivo* to further induce vascularized structures.

#### Spheroids

Various types of 3D cell aggregates, such as spheroids, organoids and tissue sprouts, have received increasing attention in regenerative medicine. Among them, spheroids break the limitation of traditional monolayer cell culture and make the connection between cells more closely, are widely used in high-throughput evaluation *in vitro* and tissue repair *in vivo* [167]. MSCs transplantation is a promising treatment for ischemia–reperfusion injury. Sun *et al*. [76] used 3D spheroid culture, enhanced the nutritional and anti-inflammatory properties of MSCs, while increasing the secretion of VEGF, which was helpful for transplantation. Cuvellier *et al*. [168] printed PHHs with GelMA and found that the spontaneous polarization of the cells produced hollow spheroids. These highly differentiated PHHs were then implanted in mice and showed the ability to be printed to the structures for vascularization. Park [77] investigated the use of photobiomodulation treatment to differentiate human adipose stem cells in spheroids and stimulate angiogenesis to improve the recovery of liver function. Such hepatic spheroids act as individual vascularized units, facilitating the development and functioning of new microvascular networks within the implanted tissue structure [77]. In terms of disease models, Suurmond *et al*. [81] proposed an *in vitro* model of nonalcoholic fatty liver disease formed by co-culture of hepatic progenitor cells (HepaRG), umbilical vein endothelial cells (HUVECs) and Kupffer cells into spheroids. Compared with those made from HepG2 cells, these spheroids made from HepaRG showed a closer trend to the functions in humans.

#### Organoids

Organoids are relatively simple to generate and have the ability to play a good role in damage repairment [169]. In addition to their application in clinical transplantation, liver organoids can be used as a salvage bridge in the transition from liver failure treatment to liver regeneration or as a supplement to extensive liver resection and temporary maintenance of the liver while awaiting transplantation. Based on this purpose, Yang *et al*. [73] used HepaRG to prepare liver tissue patches, and after 7 days of differentiation *in vitro* with DMSO, these patches were transplanted into liver-damaged mice and found that the survival time of the mice was significantly increased. New blood vessels began to form on Day 14 post-transplantation, and some common human-specific biomarkers were detected. In another research by Wu *et al*. [75], mesenchymal stromal cells (MSCs) aggregates were deposited and attached to decellularized liver scaffolds and transplanted into the omentum of liver-injured rats. This liver tissue had a stable structure, with completed functional expression and angiogenic capacity [75]. Bioinks containing a variety of hepatocyte ECM components will be used to fabricate complex liver organoids, which can promote the transformation of undifferentiated cells into hepatocytes and the expression of specific proteins. Janani *et al*. [170] utilized human adipose-derived MSCs, HUVECs and human HSCs and applied two bioinks to support parenchymal and non-parenchymal cells, hepatic lobular organoids were constructed with functional sinusoidal lumen-like networks in both horizontal and vertical orientations. The results showed that this co-cultured liver model exhibited enhanced albumin production, urea synthesis and cytochrome P450 (CPR) activity.

### Bioprinted vascularized liver models

In contrast, the strategies used by mature cells to build vascularized liver tissue are more diverse. Since cells do not have the ability to differentiate, 3D bioprinting is more conducive to the construction of liver tissue with precise vascular networks that can grow according to a preset pattern that preserves structure well and promotes cell-to-cell interactions. 3D bioprinting involves mixing cells and hydrogels together to form bioinks, and then applying additive manufacturing techniques such as extrusion, photocuring and inkjet to precisely deposit the bioinks *in vitro* to construct *in vitro* tissues and organs [171, 172]. While microvascular induction uses prefabricated cells that can self-assemble to build corresponding structures [100, 173]. Both strategies are followed by a phase of organizational remodeling and maturation [174]. In Table 3, we summarize some biofabrication methods for liver model construction.

**Table 3.** Biomanufacturing approaches for liver model construction

Biomanufacturing approaches	Advantage	Disadvantage	References
Inkjet-based bioprinting	Small tissues and organs with high resolution requirements can be constructed, ability to print low-viscosity biomaterials	Inability to provide continuous flow, low vertical structure accuracy, low cell density	[175–178]
Extrusion-based bioprinting	Broad bioink compatibility, printable with multiple viscosities, good biocompatibility, printable with high-cell densities, continuous gradient printing is possible	Low resolution, slow print speed, only suitable for viscous liquids	[70, 175, 178, 179]
Photocuring-based bioprinting	High printing resolution; can construct more complex structures; high printing speed	Toxicity of UV light sources to cells, possible damage to cells with photo-initiators	[147, 178, 180–183]

#### Inkjet-based bioprinting

**Figure 4.**
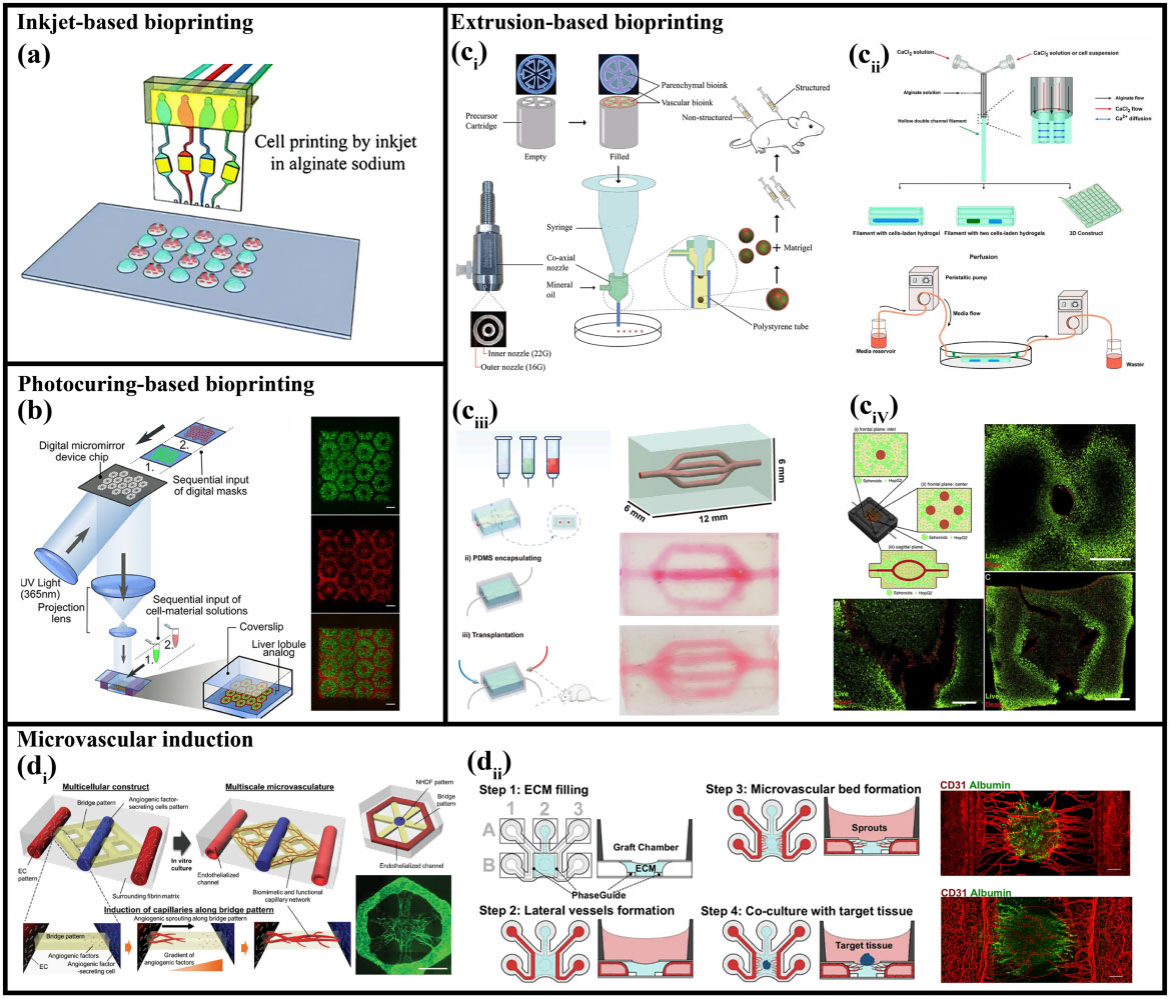
Bioprinted vascularized liver models. (**a**) Inkjet-based bioprinting. Adapted with permission from Ref. [177]. (**b**) Photocuring-based bioprinting. Adapted with permission from Ref. [189]. (**c**) Extrusion-based bioprinting. (**c_i_**) Pre-set micro-extrusion printing. Adapted with permission from Ref. [102]. (**c_ii_**) Coaxial extrusion printing. Adapted with permission from Ref. [67]. (**c_iii_, c_iv_**) Sacrificial printing. Adapted with permission from Ref. [69, 187]. (**d**) Microvascular induction. (**d_i_**) Microvascular induction to generate liver organoids. Adapted with permission from Ref. [103]. (**d_ii_**) Microvascular induction to generate hepatic spheroids. Adapted with permission from Ref. [196].

Inkjet-based bioprinting uses voltage to change the shape of piezoelectric materials and generate pressure and then eject droplets from nozzles to achieve the printing of small-scale structures, which can be widely used in materials such as living cells, biomolecules and biocompatible hydrogels [184]. Some attempts have been made to utilize inkjet-based bioprinting in vascularized liver tissue engineering. Zhang *et al*. [177] used inkjet as a cell-patterning method in microchips to form an integrated system that mimics vascularized structures by printing different kinds of cells at designed locations combined with corresponding microchannels (Fig. 4a). At the same time, the human hepatoma cell line HepG2 and the human glioma cell line U251 were co-cultured and subjected to drug metabolism and diffusion experiments. The experimental results showed that the drug was metabolized by HepG2, showing a significant anticancer effect on U251. Arai *et al*. [176] used inkjet printing to construct monolayer 3D hydrogel sheets for hepatocyte attachment. They used two gel sheets to create a sandwich structure to form parallel layers of liver cells. In this way, a hepatic cord structure can be achieved and has some potential for the formation of the vascular system. However, due to the slow printing efficiency and very few selection of low viscosity bioink of inkjet printing, also the applied voltage has the problem of affecting the specific function expression of cells, so the usage of inkjet bioprinting in liver tissue construction has limited.

#### Extrusion-based bioprinting

The extrusion-based 3D bioprinting technologies extrude the material through a nozzle into continuous filaments and uses the movement of the nozzle or the receiving plate to build different 3D structures [18]. It has attracted much attention in the preparation of liver tissue due to its ease of use and potential to adapt to various bioink viscosities, offering high cell loading densities and minimal damage to cells [185]. Meanwhile, extrusion printing enables the construction of continuous gradient tissue. Liu *et al*. [179] printed a pattern of endothelialized tissue in which four sections loaded with human dermal fibroblasts (HDFs), HepG2 human hepatocytes, human MSCs (hMSCs) and cell-free bioinks were deposited on the bottom, respectively. Then a vasculature similar to encapsulating HUVECs was integrated on the top [179].

##### Pre-set

However, extrusion bioprinting has the disadvantages of low printing resolution and slow printing speed, so it is still a challenge for building vascular structures. In order to overcome these problems, Jin *et al*. [70] proposed the pre-set extrusion technology in 2018, with the principle that the fluid with a low Reynolds number is not easy to mix. They printed a complex multimaterial high-resolution structure containing HepG2 cells and endothelial cells [70]. Two years later, the team used the technique to print vascularized structures resembling liver lobules, including the central lumen and sinusoids [101]. After culture, endothelial cells were observed to spread well on the surface and inside the lumen. Recently, combined with microfluidic emulsification technology, hepatic lobular vascularized multicellular spheroids were successfully constructed on the scale of hundreds of micrometers. In these spheroids, endothelial cells were distributed on the outside, which ensures the integrity of the overall structure, and forms a radial vascular architecture similar to the liver lobule (Fig. 4ci) [102].

##### Coaxial extrusion printing

Coaxial extrusion printing has the characteristics of simple and high practicability and is widely used in the construction of the vascular network. Pi *et al*. [186] achieved a perfusable circumferential multilayer tissue structure using a digitally tunable multilayer coaxial nozzle. Besides, Yu *et al*. [67] used dual-channel filaments for extrusion bioprinting, successfully constructed vascular access and simulated the dynamic function between hepatocytes through sequential perfusion (Fig. 4cii).

##### Sacrificial printing

Because the scale of blood vessels is relatively small, and the precision of extrusion printing is generally not enough to construct the structure of microvessels, the sacrificial printing method to forming a hollow channel structure by heating and dissolving temperature-sensitive materials is proposed and widely used in the fabrication of vascular networks. Pimentel *et al*. [69] utilized sacrificial printing to construct a tissue with an intact 3D perfusable network and soft-tissue-scale stiffness. The obtained tissue constructs were cultured by perfusion using a custom-built fluidic platform, resulting in significantly prolonged survival (>14 days) (Fig. 4civ) [69]. Liu *et al*. [187] dissolved the fugitive inks Pluronic F127 to form channels and incubated endothelial cells (ECs) to form vascular beds (Fig. 4ciii). The printed constructs can be perfused through branched endothelial vasculature to support well-formed 3D capillary networks, which then mimic mature and functional liver tissue in terms of liver-specific protein synthesis.

#### Photocuring bioprinting

The photocurable bioprinting method uses light to cross-link the bioink, and build up layer by layer with the preset pattern, the most common used manner also known as digital light processing. This printing strategy, with the advantages of higher accuracy, faster printing speed and higher resolution, helps build more complex and complete microstructures within the hepatic lobules [188]. However, this method has potential phototoxicity, which may have a certain impact on the viability of cells. Ma *et al*. [189] encapsulated hepatocytes and endothelial- and mesenchymal-derived supporting cells in complementary patterns and restored the hepatic lobular structure by constructing vascular channels through a photopolymerization method of a hydrogel matrix (Fig. 4b). Grigoryan *et al*. [190] created 3D entangled multivascular networks using photopolymerizable hydrogels. Then, a more advanced vehicle was constructed that could deliver liver aggregates in native fibrin gels with vascular compartments that could be seeded with endothelial cells. Bernal *et al*. [180] build highly complex and unique structures by reducing scattering by refractive index matching of specific intracellular components, a development that enables high-resolution volumetric bioprinting. This research opens up the possibility of constructing sophisticated microvascular networks in the future. These findings demonstrate the close relationship between a fine structure produced by photocuring methods and the resulting biological function, further underscoring the potential of biofabrication for advanced tissue engineering.

#### Photosymbiotic tissue engineering

Tissue engineering offers the possibility of *in vitro* biofabrication of three-dimensional (3D) tissues, but due to the complexity of vascularized structures, ideal 3D tissue scaffolds are difficult to achieve [191]. To overcome this difficulty, the strategy of introducing oxygen in tissue engineering has been extensively explored [192]. Given that oxygen is produced by photosynthetic microorganisms such as microalgae and cyanobacteria in nature, and they have a symbiotic relationship with a variety of eukaryotic hosts including animals, a nascent photosymbiotic tissue engineering has gradually been widely studied [193]. It has to be mentioned that Maharjan *et al*. [194] used sacrificial printing to develop an *in vitro* vascularized tissue structure with sufficient oxygen supply. Among them, they utilized algae to act as natural photosynthetic oxygen generators in the liver tissue structure, support the viability and function of HepG2 cells in the surrounding GelMA matrix, and evenly distribute HUVEC layers to form endothelialization channels. This method can effectively avoid cell death due to hypoxia, providing a new idea for *in vitro* combinatorial construction.

### Microvascular induction

Bioprinting is a well-established approach to generate large vessels embedded in liver tissues; however, the common bioprinting methods are not suitable to create microvascular due to the limitations of the printing resolution. Methods of microvascular induction target the formation of vascular channels by exploiting the tendency of cells to grow toward higher nutrient concentrations. This method can break through the limitations of printing resolution and enables the formation of microvascular systems that are closer to the physiological range [104, 195]. Following this strategy, Son *et al*. used angiogenic factor-secreting cells to create angiogenic factor gradients along a bridge pattern, using a method of biological self-assembly to form microvascular networks (Fig. 4di) [103]. However, although this method can build a network of blood vessels, the hepatocytes is not included around the network, which cannot simulate the direct interaction between blood flow and the cells. To compensate for this shortcoming, Bonanini *et al*. [196] established a vascular bed by inducing endothelial cells with endothelial growth factor. After transplantation, the hepatic microspheres spontaneously anastomosed with the microvascular bed to form a vascular network. And after 7 days of co-culture in the Disse-like structural space between hepatocytes and endothelium, endothelial cells were found to penetrate the liver microtissue and form stable, perfusable microvasculature (Fig. 4dii) [196]. Although this method of microvascular induction can construct fine capillary networks, it still has the disadvantages of small overall scale, long construction time and unstable directional formation ability.

### Microfluidics-based vascularized liver models

**Figure 5.**
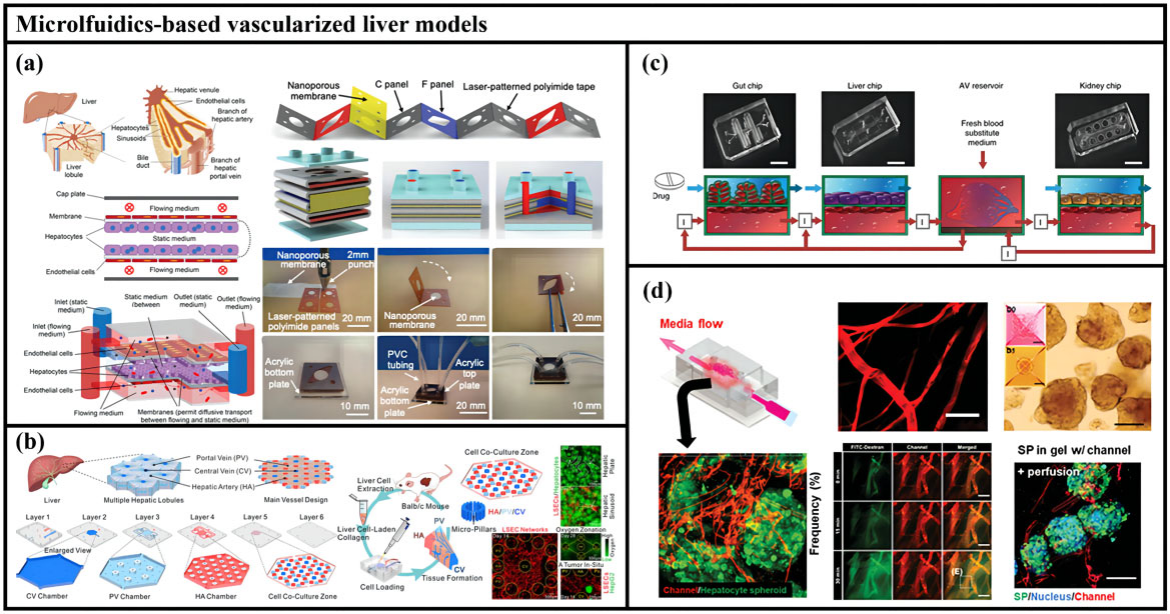
Microfluidics-based vascularized liver models. (**a**) Liver sinusoidal fold chip. Adapted with permission from Ref. [30]. (**b**) Bionic liver lobule chip. Adapted with permission from Ref. [199]. (**c**) Fluid-coupled multiorgan chip. Adapted with permission from Ref. [201]. (**d**) Nonalcoholic fatty liver organ chip. Adapted with permission from Ref. [202].

Organ-on-a-chip technology is a microfluidic-based technology, which is used to cultivate a variety of living cells in a microchamber under continuous perfusion conditions to form a biomimetic system of *in vivo* organ microenvironment [197]. Although various liver-on-chips technologies have been developed, it remains a great challenge to use this technology to simulate hepatic lobule structures that contain perfusable sinusoidal networks. Current liver-on-chips lack the ECM necessary for hepatocytes and the biliary system necessary for the excretion of bile. Lee *et al*. [198] constructed a multicell cultured 3D liver chip using liver dECM bioink. The chip has dual-flow hepatic vascular/biliary channels, overcoming the limitations of existing models and successfully observing the formation of the biliary system and enhanced liver function in the chip. Xie *et al*. [30] utilized a rigid polymer, and a soft porous membrane folded together to form a stack of three adjacent flow chambers separated by the membrane. Endothelial cells were seeded in the upper and lower chambers to simulate sinusoids, and hepatocytes were seeded in the middle chamber. Nutrients and metabolites flow through the simulated sinusoids and diffuse between the vascular channels and the hepatocyte layer, simulating physiological microcirculation (Fig. 5a) [30]. Ya *et al*. [199] developed a realistic biomimetic hepatic lobule-on-a-chip on which a perfusable sinusoidal network was realized using a microfluidic-guided angiogenesis approach. Furthermore, after self-assembly, the oxygen concentration was adjusted to mimic the physiological level of dissolution provided by actual hepatic arterioles and venules (Fig. 5b) [199]. These studies showed that, chips with vascularized structures can better simulate the biomimetic liver microstructure, have the higher metabolic capacity and longer-lasting hepatocyte functions.

Due to the high-throughput characteristics of liver-on-chips, they are often used in disease models and drug metabolism studies [200]. Lasli *et al*. [83] presented an *in vitro* system to study nonalcoholic fatty liver disease by first co-culturing HepG2 cells and HUVECs into spheroids and then transferring the spheroids to a chip system with an array of interconnected hexagonal microwells. Proven to help monitor liver function by increasing the interaction of albumin secretion with HepG2-HUVEC and increasing the production of reactive oxygen species in adipose spheroids. Drugs typically pass through the endothelium-parenchymal tissue interface in the body, and the endothelium can contribute to drug-toxic behavior. Herland *et al*. [201] designed a fluid-coupled multiorgan chip to quantitatively demonstrate the pharmacokinetic and pharmacodynamic models of human physiological drugs through drug absorption, metabolism and excretion (Fig. 5c). Using the chip can simulate the drug transfer under physiological conditions between organs through the endothelial-lined vasculature and maintain long-term viability. Besides, Lee *et al*. [202] developed a new liver model aimed at enabling the implantation and maintenance of liver buds in perfusable 3D hydrogels in which a microvascular network develops within the 200 µm diffusion limit. This system replicates inflammation, lipid accumulation, and fibrosis processes in the progression of nonalcoholic fatty liver disease and predicts outcomes in mouse models (Fig. 5d) [202]. Recently, Chhabra *et al*. [203] developed a microfluidic chip platform called a structurally vascularized liver ensemble. It enables the control of hemodynamic changes to mimic those that occur during liver injury and regeneration and supports the management of biochemical inputs such as cytokines and paracrine interactions with endothelial cells. The model can provide information that cannot be gleaned from mouse or other animal studies, allowing scientists to more precisely track the processes involved in liver regeneration.

## Vascularized liver tissue applications and evaluation

The liver has a complex and unique microenvironment with multiple cell–cell interactions and an internal vascular network. Tissues with abundant vascularization can highly restore the cellular microenvironment and help improve the secretion and metabolic functions of hepatocytes. This kind of highly biologically active *in vitro* tissue will be widely used in transplantation and regeneration. In addition, the development of clinically relevant vascularized liver models that can mimic the stimulation and induction of molecular pathways can help to elucidate disease mechanisms in biomedical research and applications in preclinical drug screening. It also provides a powerful platform for personalized liver toxicity screening [204], helping pharmaceutical companies to speed up drug development and drug toxicity analysis [3, 205]. For functional assessment of transplant regeneration, secretion of albumin and urea is the focus of attention. Cytochrome P450, transaminases and bilirubin are hallmark biomarkers in the presence of liver injury and are therefore frequently detected in disease modeling and drug screening [206]. As shown in Table 4, we exemplify the applications and evaluation indicators of some vascularized liver tissues.

**Table 4.** Application and evaluation indicators

Cell type	Culture medium^a^	Structure	Applications	Biomarkers	References
HepaRG	High Glucose Dulbecco’s Modified Eagle Medium (DMEM) with dimethyl sulfoxide (DMSO) medium	Scaffold	Transplantation	Albumin, α-1 antitrypsin, factor VII, factor IX, CYP1A2, CYP3A4, alanine aminotransferase (ALT), aspartate aminotransferase (AST), direct bilirubin (DBIL), gamma-glutamyl transpeptidase (GGT), alkaline phosphatase (ALP), albumin/globulin ration (A/G)	[73]
HepG2, HUVEC	FBS and penicillin-streptomycin solution mixed in fresh DMEM	Hepatic lobule spheroids	Transplantation	MRP2, albumin, β-catenin, P-β-catenin, α-1 antitrypsin, urea, CYP3A4, CYP1A2, CYP2B6, CD31	[102]
Mesenchymal stem cells (MSCs)	Heparinized acellular extracellular matrix and mesenchymal stem cell culture medium	Spheroids	Transplantation	albumin (ALB), aspartate aminotransferase (AST), alanine aminotransferase (ALT), total bilirubin (TIBL), FOXA2, NR1I2, SLC27A5, INS2, CYP1A1, CYP3A9, GPX3, AKR1D1	[75]
HepaRG, stellate cells (LX-2), HUVEC	William’s E medium with fetal bovine serum Hyclone III, penicillin, streptomycin, human insulin, l-glutamine, hydrocortisone hemisuccinate and DMSO	Scaffold	Liver fibrosis induction	Albumin, ACTA2, COL1A1, MMP2, TIMP1, Cytochrome P450 isoforms (CYP3A4, CYP2B6, CYP2E1, CYP2C9, CYP2C19)	[82]
Primary human hepatocytes, HSCs, HUVEC, Kuffer cells	DMSO was spiked into the TGF-β1 dosing solution and standard culture medium	Scaffold	Liver fibrosis induction	miR-122, albumin, urea, lactate dehydrogenase (LDH)	[207]
HepG2	DMEM with fetal bovine serum	Hepatic lobule organoids	Hepatocellular carcinoma	Albumin, E-cadherin, matrix metalloproteinases (MMP2 MMP9), AFP, Twist-related protein 1 (TWIST1)	[208]
Primary hepatocellular carcinoma	DMEM/F12, penicillin/streptomycin, GlutaMAX, HEPES, B27, N2, N-acetyl-l-cysteine, nicotinamide, recombinant human (Leu15)-gastrin I, recombinant human EGF, recombinant human FGF10, recombinant human HGF, forskolin, A8301, Y27632, dexamethasone	Scaffold	Hepatocellular carcinoma	Ki-67, AFP, Raf-1, VEGFR1, VEGFR2	[209]
HepaRG	William’s E medium with fetal bovine serum, l-Glutamine, recombinant human insulin, hydrocortisone hemisuccinate, penicillin/streptomycin, DMSO	Scaffold	Viral transcription	Albumin, CYP3A4, lactate dehydrogenase (LDH), adenoviral DNA	[210]
HepG2	Minimum essential medium (MEM) α supplemented with FBS and antibiotic–antimycotic	Spheroid	Drug-induced hepatotoxicity	MPT, cytosolic calcium, caspase-3	[28]
Hepatocytes, endothelial cells, Kupffer cells, and stellate cells	William’s E medium containing GlutaMAX, ITS+ Premix [human recombinant insulin, human transferrin, selenous acid, BSA, and linoleic acid], dexamethasone, ascorbic acid, fetal bovine serum, and penicillin/streptomycin	Organ-on-a-chip	Drug toxicity, predict drug-induced liver injury (DILI)	Cytochrome P450 isoforms (CYP1A, CYP2B, and CYP3A), MRP2, miR122, α-GST, keratin 18, ALT, AST, GLDH	[66]
Human adipose-derived mesenchymal stem cells (ADMSCs), HUVEC, human hepatic stellate cells (HHSCs)	HiPer high glucose Dulbecco’s Modified Eagle Medium, containing fetal bovine serum, l-glutamine, basic fibroblast growth factor, endothelial cell growth supplement, stellate cell growth supplement	Hepatic lobule organoids	Drug toxicity, drug screening	Cytochrome P450, albumin, cytokeratin 18, CYP2E1, fibronectin, HNF4α markers, α-fetoprotein (AFP)	[170]
Human pluripotent stem cell (hPSC)	Advanced DMEM/F12 supplemented with GlutaMAX, HEPES, B27, BSA, N-acetyl-L-cysteine, [Leu15]-gastrin I human, A83–01, DAPT, 3 μM dexamethasone, FGF19, BMP7 and HGF. For suspension culture, media were supplemented with Matrigel	Spheroid	Drug-induced liver injury (DILI)	Cytochrome P450, albumin, urea	[5]
HepaRG	William’s E medium supplemented with L-Glutamine, fetal bovine serum, hydrocortisone hemisuccinate, recombinant human insulin, penicillin/streptomycin and DMSO	Scaffold	Drug toxicity	lactate dehydrogenase (LDH), albumin, urea	[211]

a The culture medium in Table 4 is all the components of the co-culture medium, among which DMSO is mostly used after 1–2 weeks to induce differentiation.

### Transplant and regeneration

**Figure 6.**
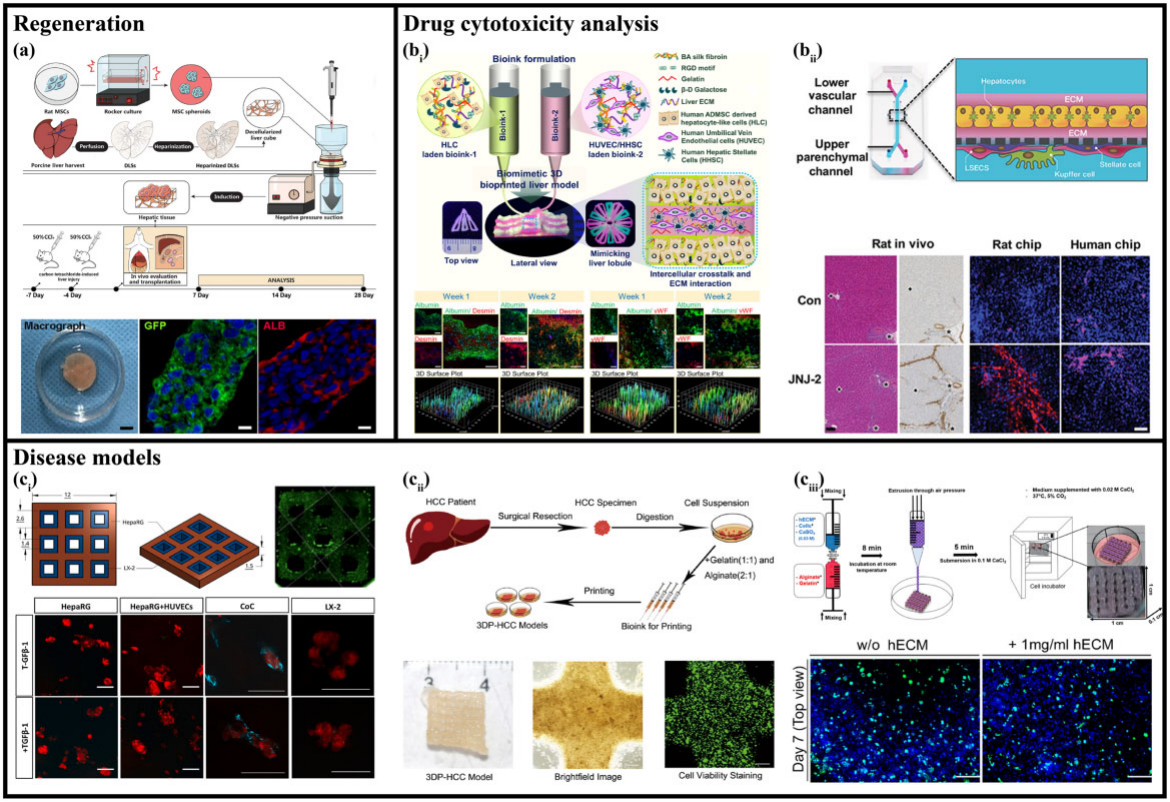
Applications of vascularized liver tissue. (**a**) Transplanted liver tissue into the liver-injured rats. Adapted with permission from Ref. [75]. (**b**) Drug cytotoxicity analysis. (**b_i_**) Vascularized hepatic lobular organoids for drug cytotoxicity analysis. Adapted with permission from Ref. [170]. (**b_ii_**) Mechanism of action of several known hepatotoxic drugs and one experimental compound. Adapted with permission from Ref. [66]. (**c**) Disease models. (**c_i_**) Liver fibrosis model. Adapted with permission from Ref. [82] (**c_ii_**) Cancer model [209]. (**c_iii_**) Virus transfection model. Adapted with permission from Ref. [210].

The shortage of organ donors is a key challenge in the treatment of end-stage organ failure, prompting the development of alternative strategies to generate organs *in vitro*. Hong *et al*. [102] subcutaneously injected structured microtissue spheroids mimicking hepatic lobules into nude mice and found the formation of functional blood vessels with structural integrity and stability. Although HepG2 is a suitable choice for *in vitro* studies of hepatic microtissues, the level of metabolic activity is low. Therefore, liver tissue composed of undifferentiated cells has better biological activity and weaker rejection response, which can improve the structural integrity and stability of implantation. Yang *et al*. [73] used HepaRG cells to construct liver organoids, acquired several liver functions after 7 days of differentiation, exhibited liver-specific protein synthesis after transplantation into liver-injured mice, and formed a functional vascular system, further supporting the substance transport function, significantly improved the survival rate of treated mice. Wu *et al*. [75] used MSCs to construct liver tissue and transplanted it into the omentum of liver-injured rats (Fig. 6a). The grafts were found to have hepatocyte-specific functions, exhibit strong proliferative activity in the ectopic liver system, and were able to anastomose the host vascular network efficiently and compatible with the host immune system.

### Disease models

The generation of various diseases is inseparable from the interaction between cell-cell and cell-ECM. Since the induction of diseases requires precise control of communication between cells, the interaction process between substances is essential. Therefore, 3D vascularized co-cultures have opened up opportunities for studying the development of hepatic disease models.

The construction of an *in vitro* hepatic fibrosis model provides a better way to study potential inducers and inhibitors of collagen expression and deposition. The findings of Norona *et al*. [207] demonstrated that Kupffer cells influence the biochemical and gene expression levels of different stages of the response and that their regulation of the injury/fibrotic response is context-dependent. Cuvellier *et al*. [82] precisely controlled cellular communication to induce liver fibrosis in 3D multicellular bioprinted constructs containing three types of cells: HepaRG, LX-2 and endothelial cells, and found fibrillar collagen deposition (Fig. 6ci). These results further demonstrate the utility of bioprinted human liver tissue for modeling and examining fibrotic events *in vitro* and understanding fibrotic injury.

In the study of liver cancer, *in vitro* liver tissue models can also play a huge role. Ma *et al*. [208] utilized a 3D biomimetic liver platform to study the behavior of various hepatoma cells in specific fibrotic settings. Xie *et al*. [209] established a patient-derived HCC (Hepatocellular carcinoma) model that retained the characteristics of parental HCC, including stable expression of biomarkers, genetic alterations and stable maintenance of expression profiles (Fig. 6cii). These models can visually and quantitatively demonstrate predicting patient-specific drug outcomes for personalized treatment. Another 3D model printed by Hiller *et al*. [210] supports efficient adenovirus replication, making them suitable for studying virus biology and developing new antiviral compounds (Fig. 6ciii).

### Drug cytotoxicity analysis

Drug development is a long, expensive and risky process with a very low success rate. Due to portal absorption of oral drugs and their high bioactivation capacity, the liver is inadvertently exposed to high concentrations of drugs and bioactive metabolites, which are often the direct cause of drug-induced liver injury. For *in situ* quantitative assessment and high-content monitoring of drug toxicity, Hong *et al*. [28] developed a HepG2 liver spheroid culture model, a promising method for screening and characterizing drug-induced hepatotoxicity. To predict drug-induced liver injury, an *in vitro* microenvironment rich in vascularization was developed. Facilitating intercellular events and crosstalk by co-culturing parenchymal and non-parenchymal cells has great advantages in modeling the complexity and diversity of drug metabolism and drug toxicity pathways. Jang *et al*. [66] applied microengineered organ-on-a-chip technology to design rat, dog and human liver-on-chips containing species-specific primary hepatocytes, kupffer cells and HSCs linked to hepatic sinusoidal endothelial cells in physiological fluid culture flow, confirming the mechanism of action of several known hepatotoxic drugs and one experimental compound. The chip detected multiple phenotypes of hepatotoxicity, including hepatocyte damage, steatosis, cholestasis and fibrosis, as well as species-specific toxicity upon treatment with the tool compounds. Janani *et al*. [170] constructed multicellular co-cultured vascularized hepatic lobular organoids for subsequent assessment of cell viability and metabolic capacity by estimating DNA concentration and lactate dehydrogenase activity after exposure to different concentrations of hepatotoxic drugs (Fig. 6bi). Cytochrome P450 activity revealed dose-dependent clinically relevant hepatotoxicity. Since a complex liver microenvironment usually causes high metabolism of drugs and toxins, cells with differentiation ability will show good results in the detection of drug toxicity. Kim *et al*. [5] generated functionally characterized liver organoids as a high-fidelity model for drug safety assessment, including high CYP450 activity and apical drug transport capacity. Schmidt *et al*. [211] prepared HepaRG cultures using alginate-gelatin-Matrigel-based hydrogels to test the toxicity of aflatoxin B1 *in vitro* (Fig. 6bi). Such organoids that restore the extracellular microenvironment may provide a suitable alternative *in vitro* for chronic hepatotoxicity studies.

## Challenges and future perspectives

Despite tremendous progress in liver tissue engineering over the past few decades, how to construct multiscale functional microvasculature with high precision and high resolution remains a challenge. Here, we summarize the current challenges and present ideas for corresponding implementations.

Firstly, in terms of vascularization accuracy, most of them are still at the millimeter level. The method of biological self-assembly can greatly improve the accuracy of formed capillary networks [104]. Micro-extrusion method can be applied to realize the alternating structure of liver tissue and vascular access and control the precision to the level of 100 microns. The *in vitro* vascularized tissue will be then formed by biological self-assembly to achieve a highly simplified liver sinusoid model. Such a capillary network, on the one hand, can promote the exchange of substances and improve the metabolic capacity of hepatocytes. On the other hand, it is also helpful for orthotopic transplantation and improves the efficiency of vascular system regeneration.

Secondly, in terms of the microenvironment, the liver has a rich variety of ECM, many types of cells, high density and relatively clear partitions, but the fabrication of a multimaterial, multicell co-culture model is a great challenge. Zhou *et al*. [212] proposed a multimaterial multiprocess fusion fabrication method that facilitates the construction of complex spatially heterogeneous structures. Combining with the embedded printing, it is possible to construct complex tissues with extra-low viscosity hydrogels such as collagen, which cannot be fabricated with traditional 3D extrusion-based bioprinting in the air [83]. A versatile embedded medium was proposed and had the ability to realize the coexistence of multiple cross-linking modes, which is helpful for the simultaneous construction of multiple materials at the same time [213, 214]. In addition, the sacrificial writing to functional tissue (SWIFT) method also helps to generate organ-specific tissue with high maturity [215]. Especially for liver tissue with high cell density and frequent material interaction, this is also an efficient construction method to create liver tissues with complex vasculatures.

Finally, in terms of functionalization, due to the lack of sources of primary hepatocytes and easy to lose functions during the cell culture process, liver tissue *in vitro* cannot restore the complete functional characterization *in vivo*. At the same time, the metabolic partitioning generated by hepatic acinus can rationally explain multiple disease models [40, 51, 54]. Therefore, microfluidic liver-on-a-chip technology can be integrated to carry out a long-term perfusion culture through the sequence of fluid flow so as to generate the gradient of metabolism, which can restore the metabolic gradient model of liver acinus. At the same time, adding more types of liver cells, such as HSCs, and Kuffer cells, can provide a more physiological model for the subsequent study of disease mechanisms and drug toxicity.

## Conclusions

In recent years, the construction of vascularized liver tissue *in vitro* has shown a new direction, and the development of this field requires a wide range of cell sources and suitable biomaterials aimed at damage repairment and drug toxicity studies. In this review, we outline various cell sources and analyze the advantages of scaffold materials for assembled cells. We first introduce the components of scaffold materials, such as protein- and polysaccharide-based natural materials, acellular extracellular matrices, synthetic polymers and nanocellulose materials. We then outline common and novel strategies for preparing vascularized liver tissues based on different types of cells. With the *in vitro* fabricated vascularized liver tissues, many biomedical applications can be applied on these models, such as liver regeneration, disease model as well as drug screening. In terms of transplantation regeneration, prevascularized tissue can be anastomosed with the host’s vascular system, increasing the success rate of transplantation. At the same time, animal experiments are ultimately limited in terms of inducing disease models and studying drug toxicity, and vascularization models can provide suitable alternatives to more realistically understand human responses to drug testing, toxicological analysis or pathological models.
